# Supplementary material for: Effect of Petiveria alliacea Extracts on Metabolism of K562 Myeloid Leukemia Cells
Source: Int J Mol Sci. 2023 Dec 13;24(24):17418. doi: 10.3390/ijms242417418 (PMC10743714; doi:10.3390/ijms242417418)
Supplement: Supplementary file 1 [file ijms-24-17418-s001.zip › ijms-2697444-supplementary.pdf]

International Journal of *Molecular Sciences*

MDPI

Table S1. List of all metabolites identified in the K562 cell lysates treated with Esperanza.

Compound	Formula	Mass	Rt (min)	Error (ppm)	Adduct	[a]CV for QC (%)	Analytical platform	DET	[b]ID Level	[c]Fold Change	[d]*p value*	[e]VIP
**Fatty acyls**												
Hydroxyoctadecatrienoylcarnitine	$C_{25}H_{43}NO_5$	438,3218	21.31	1	[M+H]$^+$	19.55	GM-LC/MS	ESI+	3	⬆	0.010ǂ	-
Hydroxyoctenoylcarnitine	$C_{15}H_{27}NO_5$	302,1966	1.62	1	[M+H]$^+$	6.01	GM-LC/MS	ESI+	3	1.75	0.009ǂ	-
Acetylcarnitine	$C_9H_{17}NO_4$	204,1233	1.59	1	[M+H]$^+$	4.23	GM-LC/MS	ESI+	3	1.29	-	1.40
Propionylcarnitine	$C_{10}H_{19}NO_4$	218,139	2.90	1	[M+H]$^+$	5.11	GM-LC/MS	ESI+	3	0.59	0.01ǂ	-
Methylglutaric acid	$C_6H_{10}O_4$	147,0657	21.95	4	[M+H]$^+$	3.30	GM-LC/MS	ESI+	3	1.25	-	1.33
**Glycerophospholipids**												
PE 20:4	$C_{25}H_{44}NO_7P$	502.2931	22.86	1	[M+H]$^+$	11.27	GM-LC/MS	ESI+	3	6.64	0.009ǂ	2.16
PC 18:3	$C_{26}H_{48}NO_7P$	518.325	21.90	2	[M+H]$^+$	9.67	GM-LC/MS	ESI+	3	⬆	0.01ǂ	
PC 18:3	$C_{26}H_{48}NO_7P$	518.325	22.13	2	[M+H]$^+$	9.50	GM-LC/MS	ESI+	3	3.37	0.009ǂ	
PI 24:0	$C_{33}H_{63}O_{13}P$	699.4056	16.42	3	[M+H]$^+$	9.77	GM-LC/MS	ESI+	3	1.43	0.009ǂ	
PG O-39:0	$C_{45}H_{91}O_9P$	829.6306	21.95	2	[M+Na]$^+$	12.65	GM-LC/MS	ESI+	4	1.97	-	1.79
PS 2-OMe,14Me-15:0	$C_{23}H_{48}NO_9P$	514.3168	21.95	6	[M+H]$^+$	4.64	GM-LC/MS	ESI+	3	1.17	-	1.79
**Indoles and derivatives**												
Indoleacrylic acid	$C_{11}H_9NO_2$	188.0708	8.91	1	[M+H]$^+$	7.93	GM-LC/MS	ESI+	3	1.36	-	1.34
Tryptophanol	$C_{10}H_{11}NO$	184.0737	1.51	3	[M+Na]$^+$	4.67	GM-LC/MS	ESI+	3	0.61	0.009ǂ	-
**Organooxygen compounds**												
Sorbose	$C_6H_{12}O_6$	180.0634	17.48	-	-	7.94	GC-QTOF-MS	-	2	1.70	0.038ǂ	-
Phosphogluconic acid	$C_6H_{13}O_{10}P$	276.0246	22.20	-	-	5.74	GC-QTOF-MS	-	2	4.68	0.019ǂ	-
Glucose	$C_6H_{12}O_6$	180.0634	17.80	-	-	4.16	GC-QTOF-MS	-	2	1.65	-	2.01
Mannitol	$C_6H_{14}O_6$	182.0790	17.78	-	-	3.98	GC-QTOF-MS	-	2	1.69	-	1.35
Gluconic acid	$C_6H_{12}O_7$	196.0583	18.41	-	-	4.49	GC-QTOF-MS	-	2	0.75	0.009ǂ	-
Myo-inositol	$C_6H_{12}O_6$	180.0634	19.57	-	-	3.67	GC-QTOF-MS	-	2	0.94	0.009ǂ	3.55
Pantothenate	$C_9H_{17}NO_5$	220.1183	4.90	2	[M+H]$^+$	14.08	GM-LC/MS	ESI+	3	0.99	-	1.31

**Carboxylic acids and derivatives**												
Serine	$C_3H_7NO_3$	105,0426	9.79	-	-	3.26	GC-QTOF-MS	-	2	2.07	0.009ǂ	3.74
Proline	$C_5H_9NO_2$	115.0633	9.49	-	-	5.81	GC-QTOF-MS	-	2	1.03	-	1.65
Alanine	$C_3H_7NO_2$	89.0477	7.54	-	-	2.94	GC-QTOF-MS	-	2	0.86	-	4.36
Glycine	$C_2H_5NO_2$	75.0320	9.15	-	-	3.20	GC-QTOF-MS	-	2	1.03	-	1.89
Methionine	$C_5H_{11}NO_2S$	149.0510	13.28	-	-	6.38	GC-QTOF-MS	-	2	1.27	0.038ǂ	-
Glutamine	$C_5H_{10}N_2O_3$	146.0691	16.20	-	-	4.17	GC-QTOF-MS	-	2	0.79	-	3.56
Tyrosine	$C_9H_{11}NO_3$	181.0739	17.65	-	-	6.55	GC-QTOF-MS	-	2	0.92	-	1.06
LactoylLeucine	$C_9H_{17}NO_4$	204.1233	1.59	1	[M+H]$^+$	4.23	GM-LC/MS	ESI+	3	1.29	-	1.40
Leucine	$C_6H_{13}NO_2$	131.0946	10.02	-	-	7.65	GC-QTOF-MS	-	2	0.96	-	1.21
Norleucine	$C_6H_{13}NO_2$	131.0946	10.32	-	-	7.49	GC-QTOF-MS	-	2	0.92	-	1.17
Fumaric acid	$C_4H_4O_4$	116.011	10.98	-	-	6.71	GC-QTOF-MS	-	2	0.54	0.009ǂ	1.51
Glutaric acid	$C_5H_8O_4$	132.0423	13.95	-	-	6.29	GC-QTOF-MS	-	2	0.60	0.009ǂ	-
Oxalic acid	$C_2H_2O_4$	89.9953	7.97	-	-	6.52	GC-QTOF-MS	-	2	1.29	0.009ǂ	1.20
Glutamic acid	$C_5H_9NO_4$	147.0532	13.72	-	-	5.06	GC-QTOF-MS	-	2	0.87	0.009ǂ	4.96
Glutathione	$C_{10}H_{17}N_3O_6S$	308.0915	2.12	1	[M+H]$^+$	5.72	GM-LC/MS	ESI+	2	1.31	0.019ǂ	3.55
Dicarboxyethyl Glutathione	$C_{14}H_{21}N_3O_{10}S$	424.1022	2.12	0	[M+H]$^+$	19.81	GM-LC/MS	ESI+	3	0.53	0.019ǂ	-
Pyroglutamic acid	$C_5H_7NO_3$	129.0426	13.32	-	-	2.69	GC-QTOF-MS	-	2	0.95	0.038ǂ	1.90
Methyl pyruvic acid	$C_4H_6O_3$	103.0395	1.54	5	[M+H]$^+$	6.71	GM-LC/MS	ESI+	2	0.61	0.009ǂ	-
Malic acid	$C_4H_6O_5$	134.0215	12.91	-	-	4.47	GC-QTOF-MS	-	2	0.57	0.019ǂ	1.62
**Nucleosides, nucleotides, and analogs**												
Guanosine monophosphate	$C_{10}H_{14}N_5O_8P$	386.0444	1.58	8	[M+Na]$^+$	7.65	GM-LC/MS	ESI+	4	0.60	0.009ǂ	-
NADH	$C_{21}H_{29}N_7O_{14}P_2$	666.1314	1.59	1	[M+H]$^+$	7.91	GM-LC/MS	ESI+	3	0.59	0.009ǂ	-
Thioinosine monophosphate	$C_{10}H_{13}N_4O_7PS$	387.0106	1.59	8	[M+Na]$^+$	15.80	GM-LC/MS	ESI+	4	0.33	0.009ǂ	-
Methylthioadenosine	$C_{11}H_{15}N_5O_3S$	297.0896	25.10	-	-	7.81	GC-QTOF-MS	-	2	0.73	0.009ǂ	-
Inosinic acid	$C_{10}H_{13}N_4O_8P$	348.0471	26.55	-	-	26.19	GC-QTOF-MS	-	2	0.41	0.009ǂ	-
Adenosine monophosphate	$C_{10}H_{14}N_5O_7P$	370.0522	1.57	0	[M+Na]$^+$	4.02	GM-LC/MS	ESI+	2	0.44	0.009ǂ	1.05
**Organonitrogen compounds**												

Sphingosine	$C_{15}H_{31}NO_2$	240.2326	20.56	1	$[M+H-H_2O]^-$	14.22	GM-LC/MS	ESI+	3	1.80	0.019ⱡ	1.31
**Diazines**												
Uracil	$C_4H_4N_2O_2$	112.0273	10.94	-	-	11.59	GC-QTOF-MS	-	2	0.83	0.038ⱡ	-
**Imidazopyrimidines**												
Hypoxanthine	$C_5H_4N_4O$	136.0385	16.54	-	-	7.93	GC-QTOF-MS	-	2	0.45	0.009ⱡ	-

* Rt: retention time; [a]CV: coefficient of variation in the metabolites in the QC samples; [b]Identification level: level 1 structure confirmed, level 2 structure probable, level 3 unequivocal molecular formula (s), level 4 exact mass; [c]Change: change in the abundance of the specified comparison calculated as (case/control); [d]*p value* ⱡ: corresponding to the *p values* calculated by the Benjamini-Hochberg false discovery rate post hoc correction (FDR < 0.05); [e]VIP: variable importance in projection; GM: global metabolomics; LC: liquid chromatography; GC: gas chromatography; QTOF-MS: quadrupole time-of-flight mass spectrometer; PC: phosphatidylcholines; PE: phosphatidylethanolamine; PG: phosphatidylglycerol; PI: phosphatidylinositol; PS: phosphatidylserine. ⬆: metabolites present only in Esperanza-treated cell lysates.

International Journal of
Molecular Sciences

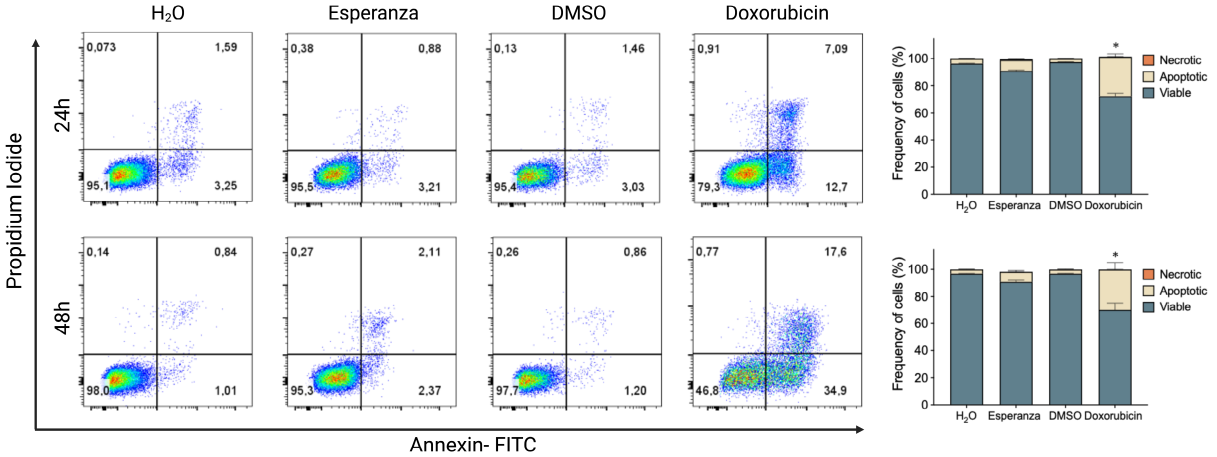

Figure S1. Cell death induction and frequency of live cells (Annexin V-, PI-), apoptotic cells (An-nexin V+, PI-; Annexin V+, PI+), and necrotic cells (Annexin V+, PI+) in K562 cells. The data from three independent experiments are presented. Significant differences were observed in the percentage of apoptotic cells after the treatment with doxorubicin compared to the rest of the treatments (yellow bars). * *$p < 0.05$*

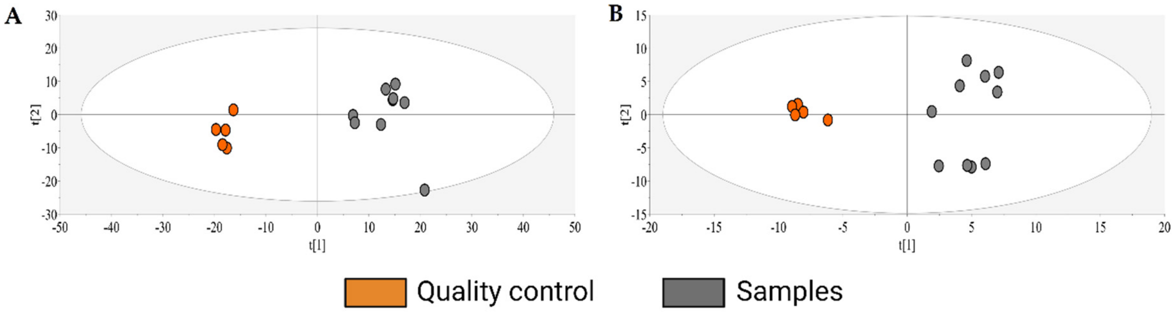

Figure S2. PCA score plots. PCA score plots for data set filtered by presence and reproducibility. A. LC-QTOF-MS, R^2: 0.825, Q^2: 0.678. B. GC-QTOF-MS, R^2: 0.54, Q^2: 0. 199.Dots in orange color denote quality control and gray dots correspond to samples (cell lysates treated and untreated).

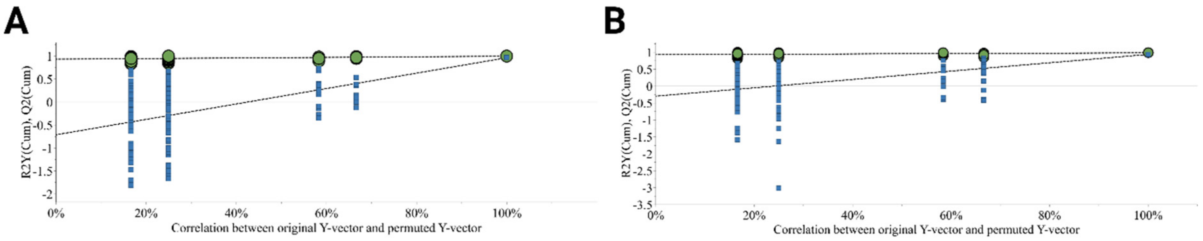

Figure S3. Permutation test. **A.** LC-QTOF-MS. B. GC-QTOF-MS.

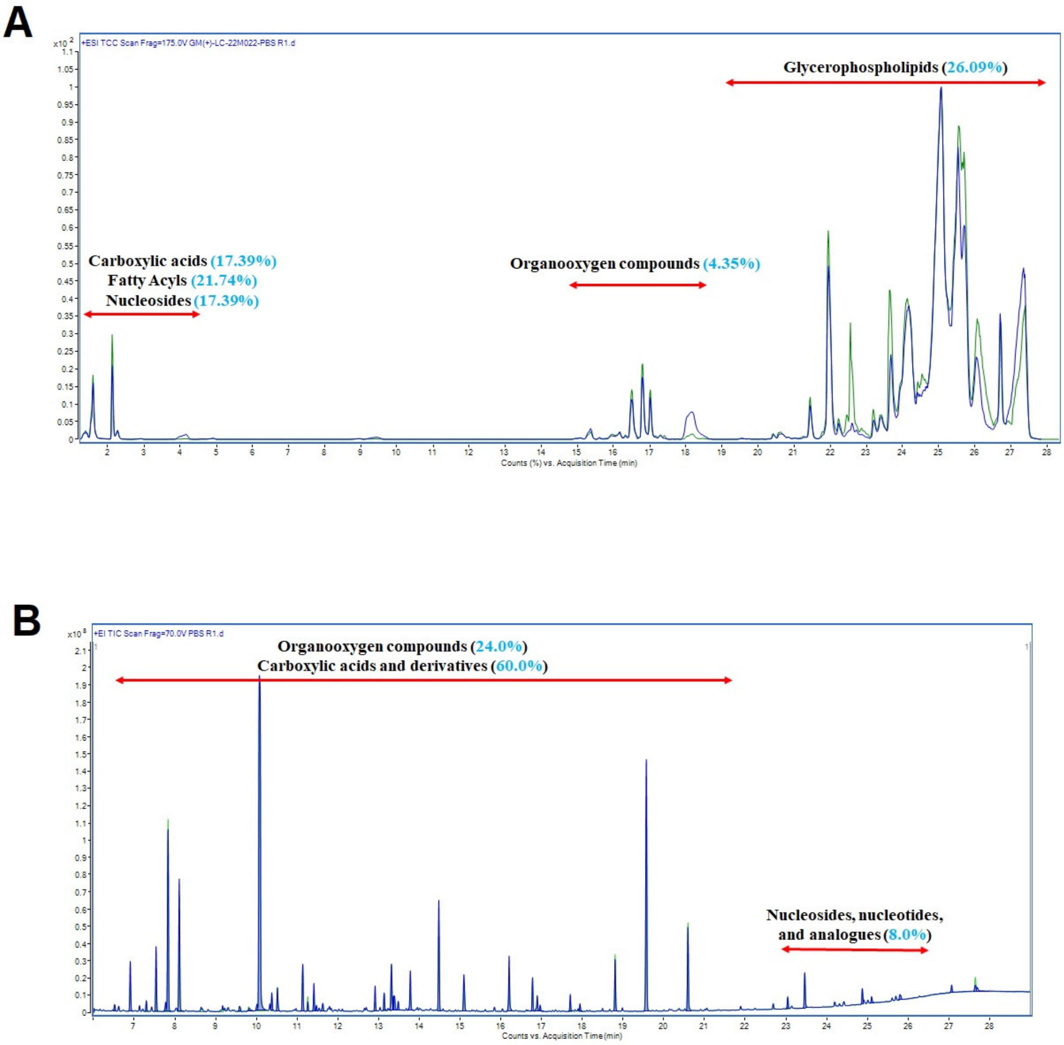

Figure S4. Comparison of LC-QTOF-MS and LC-QTOF-MS base peak chromatograms. **A.** LC-QTOF-MS for cell lysates treated with PBS (blue) and cell lysates treated with Esperanza extract (green). **B**. GC-QTOF-MS for cell lysates treated with PBS (blue) and cell lysates treated with Esperanza extract (green).
